# Antimicrobial Activity and Potential of Olive Leaf Extract as a Topical Agent to Combat *Staphylococcus aureus* and MRSA Strains: An In Vitro Evaluation

**DOI:** 10.3390/ph18091358

**Published:** 2025-09-11

**Authors:** Laura Clusa, Miriam Latorre-Millán, Ana María Milagro, Alexander Tristancho-Baró, Ana Isabel López-Calleja, Juan Manuel García-Lechuz, Blanca Fortuño, Nuno del Villar, Mario Asensio, Olga Martín-Belloso, Isabel Odriozola-Serrano, Roberto Martínez-Beamonte, Jesús Osada, Antonio Rezusta, Yolanda Gilaberte

**Affiliations:** 1 Research Group on Infections Difficult to Diagnose and Treat, Instituto de Investigación Sanitaria de Aragón, Miguel Servet University Hospital, 50009 Zaragoza, Spain; mlatorre@iisaragon.es (M.L.-M.); amilagro@salud.aragon.es (A.M.M.); aitristancho@salud.aragon.es (A.T.-B.);; 2 Research Group on Dermatology and Photobiology B59_23fD, Gobierno de Aragón, Miguel Servet University Hospital, 50009 Zaragoza, Spain; ygilaberte@salud.aragon.es; 3 Microbiology Department, Miguel Servet University Hospital, 50009 Zaragoza, Spain; jmgarcialechuz@salud.aragon.es; 4 Departamento de Bioquímica y Biología Molecular y Celular, Facultad de Veterinaria, Instituto de Investigación Sanitaria de Aragón, Universidad de Zaragoza, 50013 Zaragoza, Spainmario.asensio.franco@gmail.com (M.A.);; 5 Department of Food Technology Engineering and Science, University of Lleida, Av. Alcalde Rovira Roure 191, 25198 Lleida, Spain; 6 Agrotecnio—CERCA Center, Av. Alcalde Rovira Roure 191, 25198 Lleida, Spain; 7 Centro de Investigación Biomédica en Red de Fisiopatología de la Obesidad y Nutrición (CIBEROBN), Instituto de Salud Carlos III, 28029 Madrid, Spain; 8 Dermatology Department, Miguel Servet University Hospital, 50009 Zaragoza, Spain

**Keywords:** olive leaf extract, *Staphylococcus aureus*, MRSA, skin and soft tissue infection

## Abstract

**Background**: *Staphylococcus aureus* is one of the most prevalent bacteria in skin and soft tissue infections (SSTIs). Multidrug-resistant strain emergence, particularly methicillin-resistant *S. aureus* (MRSA), highlights the need for alternative treatments. **Objectives**: This study investigates the antimicrobial properties of olive leaf extract (OLE) and describes an epidemiological profiling of patients with SSTI who may benefit from it. **Methods**: OLE was tested in two reference strains, methicillin-susceptible *S. aureus* (MSSA) ATCC 29213 and MRSA ATCC 700699, and in 126 clinical isolates from patients with SSTIs according to Clinical Laboratory Standards Institute guidelines. **Results**: The minimum bactericidal concentration (MBC) ranged from 3.12% to 6.25% *w*/*v* for MSSA and 1.56% to 3.12% for MRSA. The lethal curve showed a reduction of 6 log_10_CFU/mL after two hours of incubation. Most of the 126 clinical samples (103 MSSA and 23 MRSA) came from skin lesions, surgical wounds, and ulcers. Over 90% of MSSA strains were resistant to less than five antibiotics, while 82% of MRSA strains were resistant to more than six. Penicillins demonstrated the lowest susceptibility rate (19.8%), whereas linezolid, daptomycin, pristinamycin, trimethoprim–sulfamethoxazole, teicoplanin, vancomycin, and OLE exhibited 100% susceptibility. No growth was observed for all clinical strains with OLE at ≥6.25% *w*/*v*. **Conclusions**: The findings suggest that OLE could become a promising alternative treatment for skin infections, particularly in the context of increasing antibiotic resistance.

## 1. Introduction

*Staphylococcus aureus* is described as a colonising or carrier-stage bacteria. It can be present on skin, nares, and mucous membranes of healthy populations. Its great ability to adapt to the stressors present in the skin leads the bacterium to cause persistent and recurrent infections [1], being responsible for more than one million deaths worldwide [2]. Additionally, it is known to be a disease promoter in inflammatory skin dermatoses as prevalent as atopic dermatitis and psoriasis [3,4]. Thus, it is one of the most frequently identified bacteria in skin and soft tissue infections (SSTIs) [5]. SSTIs range from mild infections, such as pyoderma, which are usually treated with oral antibiotics, to serious, life-threatening infections, such as necrotising fasciitis, which require intravenous treatment [6]. In SSTIs, *S. aureus* is responsible for more than thirty thousand infection-related deaths worldwide [2]; yet, there is little literature on the epidemiological profiling of this agent that could contribute to improving and to developing treatment strategies. Most studies have focused on SSTIs in general (not just those caused by *S. aureus*) [6,7] or on the methicillin-resistant *S. aureus* (MRSA) strain [8,9]. In this sense, the intensive use of antibiotics in recent decades has led to an increase in multidrug-resistant strains. In 2003, MRSA was the major pathogen in SSTIs of patients from emergency departments across the California region [10], while the MRSA USA 300 clone was the main source of community-onset *S. aureus* SSTIs in Atlanta [11]. In 2019, in Europe, more than 500,000 deaths were associated with bacterial antimicrobial resistance (AMR), with MRSA accounting for approximately 10% [12]. Nowadays, MRSA is responsible for more than 100,000 deaths worldwide and is one of the three antibiotic-resistant bacteria that cause the greatest burden of disease [5,13], hence the urgency of finding alternative and efficient methods to treat skin infections. Otherwise, infections with antibiotic-resistant strains could become the main cause of death by 2050, resulting in billions of dollars in costs [14].

Traditionally, olive trees have been broadly used in medicine [15]. In particular, olive leaf extract (OLE) has been reported to have multiple uses as a hypotensive [16,17], antioxidant [18], and hypoglycaemic [19], and its polyphenols have also been associated with reductions in the proliferation of pancreatic cancer cells and the induction of apoptosis [20]. In fact, the use of OLE is approved by the European Medicines Agency (EMA) as an oral supplement [21]. Specifically, OLE has shown in vitro antimicrobial activity traditionally attributed to polyphenols, particularly hydroxytyrosol and oleuropein [22,23], against a diverse range of microorganisms (*Candida albicans*, *Escherichia coli*, dermatophytes, *Helicobacter pylori*, methicillin-susceptible *S. aureus* (MSSA), MRSA, and anaerobic periodontal pathogens) [24,25,26,27]. It has been successfully applied in the food industry to control *S. aureus* growth during storage of Kasar cheese [28], to preserve seafood [29], to inhibit foodborne pathogens (*Listeria monocytogenes*, *E. coli*, and *Salmonella enteritidis*), and to avoid their biofilm formation [30].

Apart from that, olive tree products have been applied to the skin as anti-ageing, photoprotective, anti-inflammatory, and antioxidant agents, as well as for skin wound regeneration [31,32]. In mice, oral administration of OLE and oleuropein prevented UVB-induced skin damage [33], and intradermal injections of oleuropein accelerated skin wound healing [34]. In rats, the combination of OLE with shea butter has been proven to be effective in treating non-diabetic and diabetic wounds when infected with MRSA [35]. In humans, topical application of olive oil improved the healing of diabetic foot ulcers [36]. Furthermore, it has also been tested in clinical trials for the treatment of papillomavirus and herpes simplex [37,38], achieving faster healing for the both virus infections. Here, the present work faces two different main objectives: firstly, to test a new olive leaf extract against *S. aureus* and its resistant form, MRSA, in order to combat skin and soft tissue infections, using both standard and clinical strains; and secondly, to epidemiologically describe the *S. aureus* strains isolated from SSTIs at the Microbiology Department of the Miguel Servet University Hospital over a three-month period and characterise the number and type of resistances found in the clinical samples where OLE was tested. Hence, we define the most common type of patients and samples that could benefit from the potential use of OLE as a treatment.

## 2. Results

### 2.1. Epidemiological Description of Clinical Samples

A total of 126 clinical samples were collected and analysed over a three-month period at a Spanish tertiary hospital (Table S1). The clinical characteristics are summarised in Table 1.

**Figure 1:**
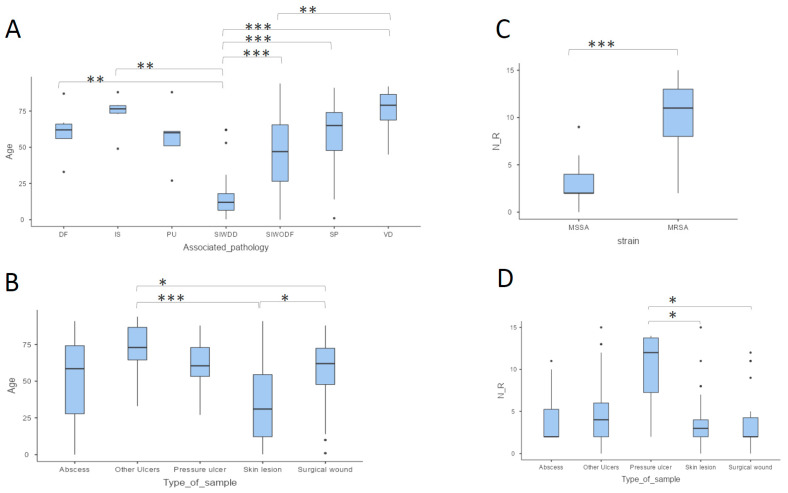
Box plots. (**A**) Age per associated pathology, (**B**) age per type of sample, (**C**) number of antibiotic resistances per strain (MSSA and MRSA), and (**D**) number of antibiotic resistance strains per type of sample. Statistically significant differences among groups are shown (* *p* value< 0.05, ** *p* value < 0.01, and *** *p* value < 0.001). DF: diabetic foot; IS: immunosuppression; PU: pressure ulcer; immobilisation; SIWDD: skin infection with dermatological disease; SIWODF: skin infection without dermatological factors; SP: surgical procedure; VD: vascular disease.

The analysis of all samples revealed that the median patient age was 56 years (IQR: 27.5–73.8). Seventy-four samples were from male patients, and 52 were from female patients. Gender showed no association with samples regarding the type of sample, location, associated pathology, age, and number of antibiotic resistances. The majority of samples came from patients with skin infections without any previous dermatological disease (31%), followed by surgical procedure samples (22.2%). They were collected predominantly from skin lesions (42.9%) and were located on the trunk (22.2%), leg (23.0%), foot (19.0%), and head (19.0%) (Table 1). Thirty-three percent of samples from patients with surgical procedures (9/27) were associated with breast cancer (Table S1). Similarly, impetigo was the most prevalent condition among samples from patients with dermatological disease, occurring in 15 out of 23 cases (65.22%). Patients with skin infection who also had a dermatological disease were significantly younger (median age: 12.0 years, IQR: 6.5–18.0) compared to those with other associated pathologies (median age range: 47.0–79.0 years) (*p* value < 0.05) (Table 1, Figure 1A). Similarly, samples from skin lesions were also obtained from younger patients (median age: 31.0 years, IQR: 12.3–54.5), compared to samples from surgical wounds (median age: 62.0 years, IQR: 47.8–72.5) or other ulcers (median age: 73.0 years, IQR: 64.5–86.8) (*p* value = 0.011 and <0.001, respectively) (Table 1, Figure 1B).

### 2.2. Antibiotic Resistance Patterns

**Figure 2:**
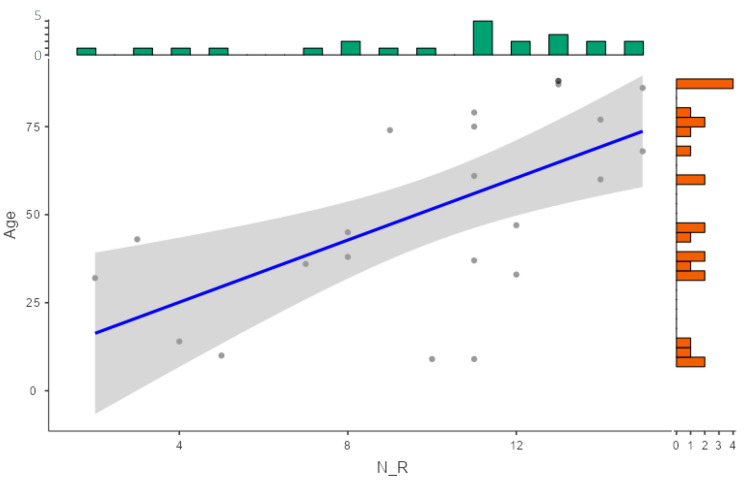
Scatter plot for age (years) versus number of antibiotic resistances (N_R) in MRSA isolates. Note: The absolute frequency of samples for each antibiotic resistance and each age is shown in green and orange respectively. The blue line shows the regression line.

**Figure 3:**
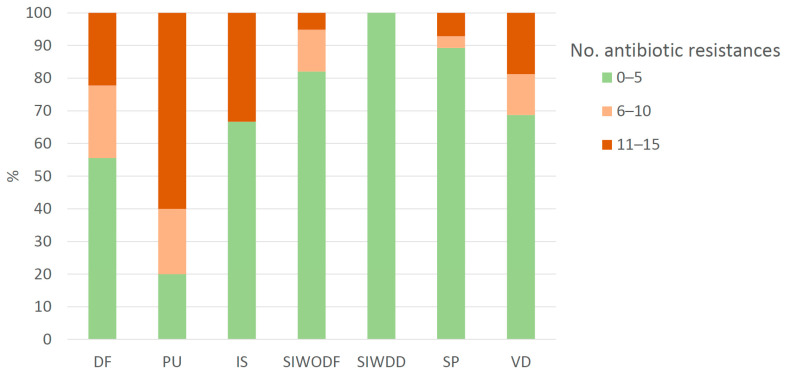
Relative frequency (%) of strains by number of antibiotic resistances classified and type of pathology. DF: diabetic foot; PU: pressure ulcer; IS: immunosuppression; SIWODF: skin infection without dermatological factors; SIWDD: skin infection with dermatology disease; SP: surgical procedure; VD: vascular disease.

**Figure 4:**
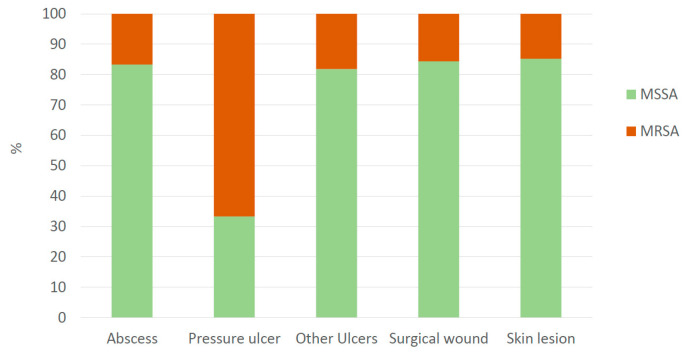
Relative frequency (%) of MSSA and MRSA strains classified by type of sample.

Of the 126 samples, 103 (82%) were identified as MSSA and 23 (18%) as MRSA, with 63 (61%) and 11 (48%) from male patients, respectively. Over 90% of MSSA strains showed less than five antibiotic resistances, whereas 82% of MRSA strains were resistant to more than six antibiotics (Table 1). As expected, the number of antibiotic resistances was statistically higher in MRSA than in MSSA (Figure 1C). In addition, a positive correlation was found between patient age and the number of antibiotic resistances in MRSA strains: the older the patient, the higher the number of antibiotic resistances (Figure 2). With regard to the overall quantity of antibiotic resistances, strains isolated from pressure ulcers showed higher resistance levels compared to those from skin lesions (*p* value = 0.034) and surgical wounds (*p* value = 0.039) (Table 1, Figure 1D). It is noteworthy that all the strains from abscesses, surgical wounds, skin lesions, and other ulcers exhibited less than five antibiotic resistances, whereas 60% of pressure ulcer isolates had more than 11 antibiotic resistances (median value: 11.0, IQR: 6.0–13.0) (*p* value < 0.001) (Table 1, Figure 3). Furthermore, a higher prevalence of MRSA was noted in these isolates compared to the rest (*p* value < 0.05) (Figure 4).

#### 2.2.1. Susceptibility Tests on ATCC (American Type Culture Collection) Samples

The susceptibility test on agar plates showed an inhibition of growth in *S. aureus* at 25, 12.5, and 6.25% *w*/*v*, whereas in MRSA, OLE was effective at 25, 12.5, 6.25, and 3.125% (*w*/*v*). The results of the broth method dilution demonstrated that the OLE MBC ranged from 3.125 to 6.25% for MSSA and from 1.56 to 3.125% (*w*/*v*) for MRSA, depending on the extraction batch and time since extraction. At least three different extraction batches were analysed. The eight clinical strains selected confirmed the MBC values, since all of them were susceptible to the last batch of OLE up to a concentration of 1.56% *w*/*v*. Notwithstanding the observed variation, all samples were effective at a concentration of 6.25% of OLE.

**Figure 5:**
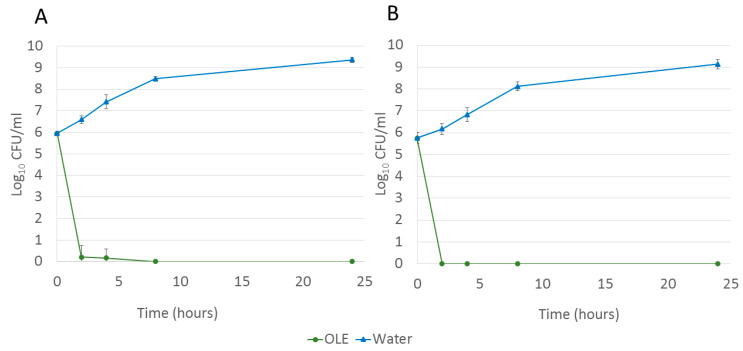
Log_10_CFU/mL after 0, 2, 4, 8, and 24 h treatment with 12.5% *w*/*v* OLE (triangle) or distilled water (circle) for (**A**) MSSA ATCC 29213 and (**B**) MRSA ATCC 700699.

Regarding the lethal curve, the concentration of OLE employed was the maximum feasible concentration (12.5% *w*/*v*), aiming for rapid bactericidal activity. After two hours, a reduction of 6 log_10_CFU/mL was achieved in both MSSA ATCC 29213 and MRSA ATCC 700699 strains treated with OLE against the control group (*p* value < 0.005). No bacterial growth (neither MSSA nor MRSA) was observed after 24 h of OLE treatment, whereas both strains reached a bacterial load of 10^9^ CFU/mL when water was used as control treatment (Table 2, Figure 5).

#### 2.2.2. Susceptibility Tests on Clinical Samples

**Figure 6:**
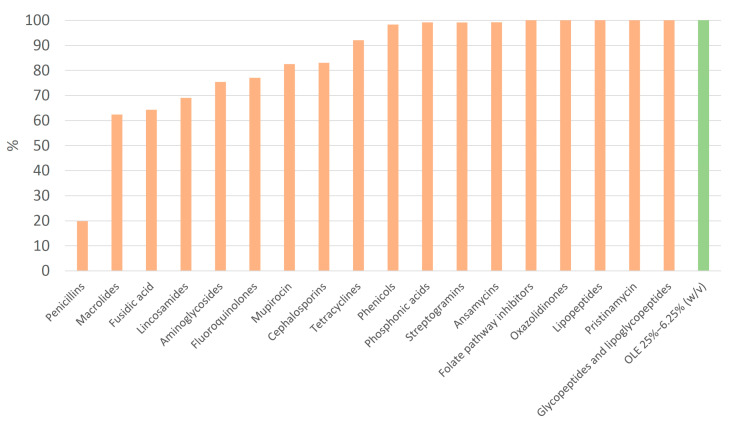
Relative frequency (%) of susceptible strains per antibiotic family. Olive leaf extract (OLE), 25 to 6.25% *w*/*v*, is shown in green.

All clinical isolates described were susceptible to OLE at concentrations of 25, 12.5, and 6.25% (*w*/*v*), with seven strains susceptible at 3.125% *w*/*v* (Table S1). All the strains were susceptible to seven antibiotics, namely ceftaroline, linezolid, daptomycin, pristinamycin, trimethoprim–sulfamethoxazole, teicoplanin, and vancomycin. Conversely, penicillin and ampicillin exhibited the highest resistance frequency (Table 3, Figure 6). As expected, all MRSA strains were resistant to beta-lactams: all penicillins and all cephalosporins except ceftaroline (Table 3). Resistance to all aminoglycosides, ciprofloxacin, levofloxacin, erythromycin, quinupristin-dalfopristin, minocycline, fosfomycin, mupirocin, and rifampicin was significantly higher in MRSA strains compared to MSSA (*p* < 0.05) (Table 3). Fluoroquinolone-resistant strains were also resistant to moxifloxacin (Table S1).

The interpretation of fusidic acid susceptibility was limited due to testing panel concentrations (2 mg/L) that did not align with EUCAST breakpoints (1 mg/L and 0.5 mg/L for topical use) [39]. Subsequently, an additional antimicrobial disc susceptibility test was conducted based on clinical characteristics and physician requests at the Microbiology department. Apart from that, up to 60% of MRSA analysed isolates were resistant to fusidic acid (Table 3). Similarly, in the case of mupirocin, the panel tests for high-level resistance (256 mg/L), which is used for short-term suppression of nasal colonisation, while topical breakpoint is 1mg/L [39].

When looking for more than one antibiotic resistance in each sample, 8 out of 126 strains were resistant to oxacillin and gentamicin. Additionally, six (three MSSA, three MRSA) out of 28 strains analysed were resistant to both fusidic acid and mupirocin. Similarly, 3 out of 28 MRSA strains tested were resistant to both fusidic acid and gentamicin. Furthermore, two of these MRSA were resistant to four antibiotics: fusidic acid, gentamicin, mupirocin, and oxacillin (Table S1).

#### 2.2.3. Antibiotic Families and Efficacy

In terms of antibiotic susceptibility by antibiotic family, penicillins had the lowest susceptibility rate (19.8%) (Figure 6), while oxazolidinones (linezolid), lipopeptides (daptomycin), pristinamycin, folate pathway inhibitors (trimethoprim–sulfamethoxazole), glycopeptides and lipoglycopeptides (teicoplanin and vancomycin), and OLE showed 100% efficacy (Figure 6).

## 3. Discussion

The present study demonstrated the antibiotic effect of OLE against multidrug-resistant strains of *S. aureus*, including the determination of their MBC and lethality curve, based on both reference and hospital clinical samples. Additionally, it provides and epidemiological description of the clinical samples analysed, hence defining the profile of patients that could benefit from treatment with OLE.

In our research, susceptibility tests on agar plates showed that MBC values were slightly lower in the MRSA ATCC strain when compared to the MSSA ATCC strain. This variation can be attributed to the faster in vitro growth rate of MSSA, which is characterised by its metabolic efficiency and adaptability. It has been reported that MSSA strains generally exhibit faster replication rates under laboratory conditions, whereas MRSA mechanisms of resistance frequently incur a metabolic cost [40,41]. Furthermore, the genetic diversity among MRSA strains can also contribute to variability in growth rates, as demonstrated by Stevens et al., who showed differences in growth speed and antibiotic tolerance in a MRSA strain [42].

Regarding other studies on OLE, Sujana et al. reported a minimum inhibitory concentration (MIC) of 0.62% for *S. aureus* with a minimum oleuropein content of 4.4 mg/mL [26]. In our case, although the amount of oleuropein was double (8.3 mg/mL in the 25% OLE) (Table S2), the MBCs were slightly higher, being 6.25% for MSSA and 3.125% for MRSA, similar to the MBC of 3.12% for MRSA described by Elnahas et al. [35]. These differences may be attributed to the extraction method or the solvent used, which could lead to a different chemical profile. Sudjana et al. employed fresh leaves, whereas we used powdered dried leaves. Markin et al. reported a MBC of 0.6% *w*/*v* for *S. aureus* from powdered leaves, but they stored their extract for up to eight weeks [25], while our OLE was used after being frozen at −80 °C for six months. Borjan et al. recommended ethanol/ethyl acetate extraction to capture key phenolic and fatty acid [22], but Pereira et al. did not see differences when using water as solvent [23]. Thus, we selected water as the solvent for our study to ensure safety and prevent interfering compounds, such as ethanol, from affecting the results by hindering the bactericidal effect. On the other hand, the majority of studies have reported the antimicrobial effect of OLE after a 24 h incubation period [26,27,30]. In our research, the OLE was able to reduce the number of viable bacteria to nearly zero after two hours of incubation, consistent with the findings of Markin et al. [25], but we also tested a MRSA ATCC strain.

Most authors assign the antimicrobial activity of OLE to polyphenols, such as oleuropein and its derivatives [22,23,43,44]. The amount of oleuropein is commonly used as a standard of OLE characterisation [26]. On the other hand, oleanolic acid has been shown to cause cell membrane damage in *L. monocytogenes*, *Enterococcus faecium*, and *Enterococcus faecalis* [45]. Olive leaf extract has also been reported to suppress flagella production in *L. monocytogenes* [30], and polyphenols from olive mill wastes showed inhibition of *E. coli* biofilm formation [46]. In our study, the short time–kill kinetics could suggest membrane permeabilization, since many plant essential oils possess antimicrobial activity [47]; however, more essays should be performed to identify OLE’s mechanism of action.

Apart from the lack of knowledge about how it works, its antimicrobial efficacy is believed to stem from the synergistic effects of its constituent compounds [23]. Liu et al. reported greater inhibition of bacterial growth when using the olive leaf extract compared to oleuropein and verbascoside separately [30]. Furthermore, Lim et al. (2016) showed enhanced ampicillin efficacy when combined with OLE, highlighting its potential to mitigate antibiotic resistance development through a multi-compound mechanism [48,49]. In our study, the efficacy of OLE was evaluated against a range of clinical strains of *S. aureus*, including those exhibiting diverse antibiotic resistance profiles. Even the most resistant MRSA strains were susceptible to OLE at three different concentrations (Table S1).

Regarding skin and soft tissue infections, the short study period and the geographic restriction could limit generalisability by misrepresenting the results in terms of seasonality and location. Despite this, the results are consistent with other unbiassed epidemiological studies: 31% of our samples were derived from a skin infection without any underlying dermatological disease, such as burns, wound infections, trauma, etc. (Table 1). Impetigo accounts for the 65% of samples collected from patients with a dermatological disease (Table S1), identified by Habib and Qadir as the most predominant infection [9]. On the other hand, the frequency of MRSA found in SSTIs varied between studies. In our research, 18.3% of isolates were MRSA (Table 1), in line with the Spanish prevalence of 20–30% [12], far from the 54.1% reported in Pakistan [9]. However, a higher proportion of MRSA (66.6%) was found in pressure ulcer samples (Figure 3), similar to the 59% reported by Manzur et al. [8]. Furthermore, the positive correlation between number of MRSA samples and age could be explained by some factors that are more likely to be present in elders, such as comorbidities or prior antibiotic use, highlighting the potential benefit of OLE use in this patient profile.

The most common treatment for *S. aureus* infection involves oral antibiotic therapy or parenteral treatment for severe cases [5,7], as well as for MRSA infection, including a wide range of adverse reactions [50]. At the topical level, the most frequent antibiotics are fusidic acid or mupirocin [51]. Frazee et al. (2005) proposed the use of trimethoprim/sulfamethoxazole, clindamycin, or vancomycin for the treatment of skin and soft tissue infections in communities with high MRSA prevalence [10]. In contrast, Habib and Qadir (2022) recommended rifampicin, minocycline, amikacin, and clindamycin for MRSA and reserving linezolid, teicoplanin, and vancomycin for severe cases [9]. We reported only seven antibiotics, apart from OLE, demonstrating susceptibility across all strains (ceftaroline, linezolid, daptomycin, pristinamycin, trimethoprim–sulfamethoxazole, teicoplanin, and vancomycin) (Table 3). In comparison to previous studies [9,10,52,53], the data presented here showed higher rates of antibiotic resistance, especially to fluoroquinolones, clindamycin, gentamicin, fusidic acid, and mupirocin. In our research, MSSA showed higher resistance rates for clindamycin (27.2%), fusidic acid (22.2%), and mupirocin (12.6%) in comparison to the 8.8%, 10.9%, and 1.4%, respectively, reported by Zhanel et al. (2021) [53]. Similarly, the MRSA resistance rates for levofloxacin (78.9%), ciprofloxacin (73.9%), clindamycin (47.8%), fusidic acid (60%), and mupirocin (39.1%) were higher than the 43.2% reported by Frazee et al. (2005) for levofloxacin or the 67.1%, 9.5%, 10.6%, and 14.1% reported by Zhanel et al. (2021) for ciprofloxacin, clindamycin, fusidic acid, and mupirocin, respectively [10,53] (Table 3). Although the susceptibility rate to fusidic acid in our research may be overestimated, as only 28 strains were analysed due to the limitations of the technique, the contrary may apply to mupirocin, since more resistant strains may be underestimated due to the concentration tested.

Looking in detail at topical antibiotic resistance, up to 22% of samples analysed were resistant to both fusidic acid and mupirocin simultaneously, which could lead to the failure of topical treatment and progression to more severe disease. Additionally, two strains analysed were found to be resistant to the four conventional topical antibiotics (fusidic acid, gentamicin, mupirocin, and oxacillin), whereas all these aforementioned strains were susceptible to OLE (Table S1). Thus, even strains resistant to multiple topical drugs were inhibited by OLE.

In addition, OLE and its derivatives have already been successfully used on the skin as protective and antioxidant agents to reduce skin damage, as well as for healing diabetic foot ulcers and even in virus infections [31,32,33,34,37,38]. Though our extract has not yet been proven as a topical clinical treatment, different formulation strategies of OLE have been successfully applied to inhibit bacterial infection or enhance wound healing in rats, such as hydrogel [32], 5% OLE ointment [54], and OLE-based ointment (with shea butter) [35], some of them having a similar concentration.

For these reasons, OLE may be used not only as treatment but also to improve healing, prevent skin infections caused by *S. aureus,* and enhance barrier repair, such as in breast cancer wounds (33% of our samples from surgical procedures) and in patients with atopic dermatitis [3]. In addition, the application of OLE could be useful in patients with comorbidities, where parenteral administration of antibiotics is ineffective due to the difficulty of reaching the local niche of the bacteria. It could also be applied in patients with antibiotic restrictions like children—indeed, the median age of patients with a skin infection and dermatological disease is 12 years old (Table 1), of which 65% are impetigo cases—thus reducing the spread of this highly contagious paediatric condition.

The potential of OLE as a topical antimicrobial is highlighted by the present work. We propose a localised treatment with OLE as single agent or in combination with other antibiotics to prevent the dispersion of bacteria. It would be easy to implement in the clinic, given that OLE is already sold as a natural supplement. For instance, it could be useful in countries with high MRSA prevalence [12], taking advantage of the lack of side effects and cost-effective treatment. However, though clinical strains were analysed, our results include only in vitro tests. Further research with animal or tissue models is needed to elucidate its clinical applicability in a real-world setting, in terms of determining its mechanism of action, stability, formulation strategy, lack of toxicity, skin penetration levelm and efficacy, alone or in combination with other antimicrobials, and in clinical trials involving patients with skin and soft tissue infections.

The present work adds epidemiological data on clinical samples and measures MBC and rapid kill kinetics. This is the first report demonstrating that its antimicrobial action, after 2 h incubation, leads to a 6-log reduction in the bacterial load (no growth observed) of both MSSA and MRSA. Additionally, it has been tested on different strains of *S. aureus*, with varying levels of antibiotic resistances. Moreover, a clinical description of all the *S. aureus* strains isolated from patients with skin and soft tissue infections in our department was conducted, identifying the most common antibiotic resistance patterns and defining the patient profile that could benefit from the potential use of OLE as a treatment.

## 4. Materials and Methods

### 4.1. OLE Extraction

Olive leaf extract was prepared as follows: *Olea europaea* trees, from a variety of olive cultivars (Empeltre, Arbequina, Picual, Hojiblanca, Cornicabra, Manzanilla, Gordal, and Sevillana) located in the municipality of Gallur (Zaragoza, Spain), polygon 11, plot 59, were selected. The leaves were harvested in March, concurrently with pruning, and manually selected to ensure the absence of diseased leaves, abnormal coloration, or any discernible morphological defect. The leaves were then dried at 65 °C, ground, and stored in an opaque container under nitrogen atmosphere until use. To prepare the OLE, the dried leaves were weighed on a precision balance and diluted in bidistilled and sterile water at room temperature to a concentration of 25% *w*/*v* (250 mg/mL). Once the dilution was prepared, it was homogenised with two zirconium/silica beads in a Mini Beadbeater 16 cell disruptor (BioSpec^®^, Bartlesville, OK, USA) at 3450 rpm for 2 min. The extract was then subjected to an ultrasound bath for 3 min at 37 °C. The supernatant was separated from the leaf debris by centrifugation at 5000 rpm for 2 min. The aqueous phases of all the fractions were pooled and centrifuged again. Finally, the olive leaf extract was filtered through a 0.22 µm filter and stored in sterile Eppendorf tubes. Once the extract was obtained, the aliquots were kept frozen at −80 °C until use. The extracts were frozen for six months without affecting their antimicrobial activity (as confirmed by tests conducted in our lab). The final concentration of the OLE was 25% *w*/*v*; this extract was diluted in sterile water to obtain the remaining concentrations: 12.5%, 6.25%, and 3.125% *w*/*v*.

### 4.2. OLE Phenolic Profile

OLE was homogenised with 70% ethanol in Milli-Q water (1600 rpm, 2 min) using an Ultra-Turrax T25-Basic mixer (Janke & Kunkel, Staufen, Germany). Then, the mixture was centrifuged (12,500 rpm, 4 °C, 15 min) and the supernatant was filtered through a 0.45 μm membrane. The resulting extracts were stored at −18 °C in the dark until further analysis. Identification and quantification of the individual phenolic compounds were carried out by UPLC-MS/MS on an AcQuity Ultra-Performance™ liquid chromatography/tandem mass spectrometry system (Waters, Milford, MA, USA) similarly to that described by Martínez-Beamonte [55]. Calibration curves with an R^2^ value of 0.99 or higher were generated using commercial standards (Sigma-Aldrich, Madrid, Spain) to ensure accurate quantification. The phenolic profile of OLE is presented in Table S2.

### 4.3. Bacteria

#### 4.3.1. Reference *S. aureus* Strains

The reference strains used were MSSA ATCC 29213 and MRSA ATCC 700699, cultured on Columbia blood agar (CBA) (Thermo Scientific™, Waltham, MA, USA) under aerobic conditions at 35 °C for 18–24 h to obtain fresh isolated colonies. These strains were used to test the bacterial susceptibility to OLE on agar plates and calculate the minimum bactericidal concentration (MBC) and the lethal curve.

#### 4.3.2. *S. aureus* Strains from Clinical Samples

This study included all samples from patients with skin and soft tissue infections that were received between 29 May and 29 August 2024 at the Microbiology Department of Miguel Servet University Hospital (a tertiary hospital whose catchment area covers approximately 400,000 people), in which *S. aureus* was isolated. Identification was performed by Matrix-Assisted Laser Desorption/Ionisation Time of Flight (MALDI-TOF) (Bruker Daltronik GmbH^®^, Bremen, Germany). These isolated strains were used to establish their antibiotic susceptibility profile and to test the bacterial susceptibility to OLE on agar plates, which may be considered the most skin-like basic model, with a nutrient-rich surface and bacteria growing on the outer layers of the agar. Furthermore, four MSSA and four MRSA with different origins and resistance patterns (*S. aureus* 001, *S. aureus* 025, *S. aureus* 045, *S. aureus* 051, *S. aureus* 008, *S. aureus* 032, *S. aureus* 050, and *S. aureus* 098, available in Table S1) were randomly selected and used to confirm the MBC value.

Data related to antibiotic susceptibility, sample type, location of infection, associated pathology (when possible), and patient age and gender were collected for each sample. The type of sample was classified according to the standard laboratory practice as an abscess, skin lesion, surgical wound, ulcer, or pressure ulcer. The location was categorised as generalised (all body), head, hand, arm, trunk, leg, foot, and toenail. The associated pathology was divided into six groups as follows: (a) immunosuppression (samples associated with angiosarcoma, basal cell carcinoma, and squamous cell cancer); (b) surgical procedure (samples from catheter, surgery, amputation, and prostheses); (c) skin infection without dermatological factors (burns, insect stings, omphalitis, knife cuts, trauma, and any skin or wound infection); (d) skin infection with dermatological disease (epidermolysis bullosa, herpes zoster, impetigo, psoriasis, and scalded skin syndrome); (e) vascular disease (elephantiasis, lymphedema, lymphocele, and vascular ulcer); (f) pressure ulcer (immobilisation); and (g) diabetic foot.

### 4.4. OLE Bacterial Susceptibility Test on Agar Plate

The inoculum was prepared by direct saline suspension of freshly isolated colonies, adjusting the turbidity to 0.5 McFarland using a MicroScan turbidity metre (Beckman Coulter^®^, Brea, CA, USA), thus containing approximately 1–2 × 10^8^ CFU/mL (colony-forming units). A sterile cotton swab was placed in the bacterial suspension and streaked in three directions over the surface of the Müller–Hinton agar (MHA) (Oxoid™, Basingstoke, UK) to obtain uniform growth. Then, 10 µL of the OLE was added on the surface, with sterile water as a negative control. The concentrations of OLE tested were 25% *w*/*v*, 12.5% *w*/*v*, 6.25% *w*/*v,* and 3.125% *w*/*v*. Plates were incubated at 35 °C for 24 h [56]. The categorisation of strains as either susceptible or resistant was determined by the presence or absence of growth. For the clinical samples, at least two different aliquots of OLE were tested.

### 4.5. Minimum Bactericidal Concentration (MBC)

The MBC of OLE was determined for the two reference strains by the broth microdilution method according to the Clinical Laboratory Standards Institute guidelines [57] with little variation. In addition, to compare the MBC values obtained on ATCC, one batch of OLE was also tested across eight different clinical strains described above. An inoculum of 0.5 McFarland was prepared as described above. The inoculum was diluted in Cation-Adjusted Müller–Hinton Broth (CAMHB) (Becton Dickinson^®^, Singapore) to achieve a concentration of 1 × 10^6^ CFU/mL. Then, 50 µL of the inoculum was incubated with 50 µL of OLE serial dilutions to a final concentration of approximately 5 × 10^5^ CFU/mL per tube. The final concentrations of OLE tested were 12.5, 6.25, 3.12, 1.56, and 0.78% (*w*/*v*). The tubes were incubated for 18–20 h at 35 °C. After that, 100 µL was spread on CBA plates and incubated again after 24 h. The MBC was the dilution at which no growth was observed in each strain. Three different batches of OLE were tested.

### 4.6. Lethal Curve

The protocol was similar to that described for the MBC. In this case, 50 µL of the inoculum was mixed with either 50 µL of 25% *w*/*v* OLE or 50 µL of sterile water (negative control) and incubated at 35 °C for 2, 4, 8, and 24 h. Subsequently, the entire contents of the tubes were spread on CBA plates, and the colonies were counted after 24 h incubation period. Regarding the inoculum, 10 µL of a 1/10,000 dilution of the inoculum was spread over the surface of a blood agar plate; the presence of approximately 100 colonies indicated an initial inoculum density of 1 × 10^8^ CFU/mL, corresponding to 5 × 10^5^CFU/mL in each assay. Additionally, the negative control for each time point was also diluted, with 10 µL used for colony counting. A minimum of six replicates, comprising two different extract batches, were conducted for each strain.

### 4.7. Antibiotic Susceptibility

Antibiotic susceptibility testing was performed using a Microscan Walkway™ semi-automatic system (Beckman Coulter^®^, Brea, CA, USA), Pos MIC panel type 33 with the following antibiotic and dilution (µg/mL) ranges according to the manufacturer (https://www.beckmancoulter.com/es/products/microbiology/conventional-panels#/, accessed on 1 December 2024): amikacin (8–32), amoxicillin/clavulanic acid (4/2–8/4), ampicillin (0.25, 4–8), ceftaroline (0.5–1), chloramphenicol (8–16), ciprofloxacin (1–2), clindamycin (0.25–2), daptomycin (1–4), erythromycin (0.5–4), fosfomycin (32–64), fusidic acid (2), gentamicin (1–8), imipenem (4–8), levofloxacin (1–4), linezolid (1–4), minocycline (1–8), moxifloxacin (0.5–1), mupirocin (256), nitrofurantoin (32–64), norfloxacin (4–8), oxacillin (0.25–2), penicillin (0.12–0.25, 8), pristinamycin (1–2), rifampin (0.5–2), synercid (quinupristin–dalfopristin) (1–4), teicoplanin (1–16), tetracycline (1–8), tobramycin (1–8), trimethoprim/sulfamethoxazole (2/38–4/76), and vancomycin (0.25–16). For some samples, only a disc diffusion susceptibility test was performed, using discs of fusidic acid (10 µg), cefoxitin (30 µg), ciprofloxacin (5 µg), clindamycin (2 µg), erythromycin (15 µg), gentamicin (10 µg), linezolid (10 µg), mupirocin (200 µg), rifampicin (5 µg), tetracycline (30 µg), oxacillin (1 µg), and trimethoprim–sulfamethoxazole (25 µg). According to EUCAST [58] the antimicrobial susceptibility was categorised as S (susceptible), I (susceptible, increased exposure), and R (resistant) for each antibiotic tested based on the clinical cut-off value [39]. For antibiotics not included (fosfomycin, cloramphenicol), epidemiological cut-offs were used [59].

### 4.8. Statistical Analysis

Values were expressed as means and standard deviations (SDs) or median values (P25–P75). Normality was tested by the Shapiro–Wilk test. Differences between the experimental groups in terms of the number of colony-forming units in the lethal curve were assessed using the Students’ *t*-test or Mann–Whitney U test depending on normality. Differences in clinical samples regarding the sample type, pathology, and location, as well as the number of antibiotic resistances and age, were analysed using the Kruskal–Wallis test. The association between age and the number of antibiotic resistances was evaluated with Pearson’s correlation coefficients through linear regression. Χ^2^ tests were used to analyse antibiotic susceptibility differences between MSSA and MRSA. Two-tailed *p* values were considered statistically significant when *p* < 0.05. All analyses and graphs were carried out with the free based R software Jamovi 2.2.5 [60], MS Excel 2016 (Microsoft, WA, USA), MS PowerPoint 2016 (Microsoft, WA, USA), and MS Word 2016 (Microsoft, WA, USA). Images for the visual abstract were downloaded from freepik (https://www.freepik.es/, accessed on 16 December 2024) and pixabay (https://pixabay.com/es/, accessed on 16 December 2024).

## Figures and Tables

**Table 1:** Descriptive characteristics of MSSA, MRSA, overall samples, age, and quantity of antibiotic resistances: age range, number of antibiotic resistances, sample type, location, and associated pathology.

	MSSA N (%)	MRSA N (%)	Overall Samples N (%)	Age (Median (P25–P75))	Quantity of Antibiotic Resistances (Median (P25–P75))
	103 (81.7)	23 (18.3)	126	56.0 (27.5–73.8)	3.0 (2.0–5.0)
Gender					
Men	63 (61.2)	11 (47.8)	74 (58.7)	59.5 (25.3–72.8)	3.5 (2.0–5.0)
Women	40 (38.8)	12 (52.2)	52 (41.3)	50.0 (28.5–74.8)	3.0 (2.0–5.0)
Age (years)					
0–18	22 (21.4)	4 (17.4)	26 (20.6)	8.0 (5.0–12.8)	3.5 (2.0–4.0)
19–65	44 (42.7)	10 (43.5)	54 (42.9)	50.0 (36.3–58.8)	3.5 (2.0–5.0)
>65	37 (35.9)	9 (39.1)	46 (36.5)	78.5 (72.3–85.3)	2.0 (2.0–4.75)
Number of antibiotic resistances					
0–5	97 (94.2)	4 (17.4)	101 (80.2)	56.0 (20.0–72.0)	2.0 (2.0–4.0)
6–10	6 (5.8)	5 (21.7)	11 (8.7)	45.0 (35.0–59.0)	7.0 (6.0–8.5)
11–15	0 (0)	14 (60.9)	14 (11.1)	71.5 (50.3–84.3)	12.5 (11.0–13.8)
Type of sample					
Abscess	10 (9.7)	2 (8.7)	12 (9.5)	58.5 (27.8–74.3)	2.0 (2.0–5.25)
Pressure ulcer	2 (1.9)	4 (17.4)	6 (4.8)	60.5 (53.3–73.0)	12.0 (7.25–13.8)
Other Ulcers	18 (17.5)	4 (17.4)	22 (17.5)	73.0 (64.5–86.8)	4.0 (2.0–6.0)
Surgical wound	27 (26.2)	5 (21.7)	32 (25.4)	62.0 (47.8–72.5)	2.0 (2.0–4.25)
Skin lesion	46 (44.7)	8 (34.8)	54 (42.9)	31.0 (12.3–54.5)	3.0 (2.0–4.0)
Location					
Generalised (all body)	2 (1.9)	0 (0)	2 (1.6)	3.5 (3.25–3.75)	4.5 (4.25–4.75)
Arm	8 (7.8)	2 (8.7)	10 (7.9)	59.0 (40.3–69.5)	3.50 (2.0–5.0)
Hand	3 (2.9)	2 (8.7	5 (4.0)	50.0 (22.0–68.0)	5.0 (4.0–5.0)
Head	21 (20.4)	3 (13.0)	24 (19.0)	50.5 (18.8–73.3)	4.0 (2.0–4.0)
Trunk	21 (20.4)	7 (30.4)	28 (22.2)	58.0 (30.0–68.0)	2.5 (2.0–6.25)
Leg	26 (25.2)	3 (13.0)	29 (23.0)	64.0 (24.0–82.0)	2.0 (2.0–4.0)
Foot	20 (19.4)	4 (17.4)	24 (19.0)	62.5 (50.0–80.5)	3.0 (2.0–6.0)
Toenail	2 (1.9)	2 (8.7)	4 (3.2)	34.0 (28.0–39.5)	2.0 (1.5–3.25)
Associated pathology					
Diabetic foot	7 (6.8)	2 (8.7)	9 (7.1)	62.0 (56.0–66.0)	4.0 (2.0–6.0)
Immunosuppression	4 (3.9)	2 (8.7)	6 (4.8)	76.5 (73.5–78.8)	3.5 (2.25–9.25)
Immobilisation (pressure ulcer)	2 (1.9)	3 (13.0)	5 (4.0)	60.0 (51.0–61.0)	11 (6.0–13.0)
Skin infection *w*/*o* dermatological factors	31 (30.1)	8 (34.8)	39 (31.0)	47.0 (26.5–65.5)	2.0 (2.0–4.0)
Skin infection with dermatological disease	22 (21.4)	1 (4.3)	23 (18.3)	12.0 (6.50–18.0)	4.0 (2.0–4.0)
Surgical procedure	25 (24.3)	3 (13.0)	28 (22.2)	65.0 (47.8–74.0)	2.0 (2.0–4.0)
Vascular disease	12 (11.7)	4 (17.4)	16 (12.7)	79.0 (68.8–86.5)	4 (2.75–6.5)

Note. Data are expressed as absolute (N) and relative (%) frequencies for categorical variables, and as medians and interquartile ranges (M (P25–P75)) for continuous ones. MSSA: methicillin-susceptible *Staphylococcus aureus*; MRSA: methicillin-resistant *Staphylococcus aureus*; *w*/*o*: without.

**Table 2:** MSSA ATCC 29213 and MRSA ATCC 700699 concentration after 0, 2, 4, 8, and 24 h, treated either with 12.5% *w*/*v* OLE or with distilled water (control).

	MSSA		MRSA	
	Control	OLE	Control	OLE
0 (hours)	5.95 ± 0.11	5.95 ± 0.11	5.76 ± 0.25	5.76 ± 0.25
2 (hours)	6.60 ± 0.18	0.22 ± 0.53	6.17 ± 0.26	0.00 ± 0.00
4 (hours)	7.42 ± 0.33	0.17 ± 0.41	6.83 ± 0.33	0.00 ± 0.00
8 (hours)	8.49 ± 0.10	0.00 ± 0.00	8.12 ± 0.20	0.00 ± 0.00
24 (hours)	9.37 ± 0.13	0.00 ± 0.00	9.14 ± 0.22	0.00 ± 0.00

Note. Data report means and standard deviations (SDs) of log_10_CFU/mL of six replicates. OLE: olive leaf extract; MSSA: methicillin-susceptible *Staphylococcus aureus*; MRSA: methicillin-resistant *Staphylococcus aureus*.

**Table 3:** Antibiotic susceptibility characterisation for overall analysed and resistant isolates, including MSSA and MRSA.

Antibiotic Class	Antibiotic	Overall N	Resistant N (%)	MSSA N (%)	MRSA N (%)	*p* Value
Fucidane	Fusidic acid	28	10 (35.7)	4 (22.2)	6 (60.0)	
Aminoglycosides		126	31 (24.6)	17 (16.5)	14 (60.9)	<0.001
	Amikacin	119	16 (13.4)	3 (3.0)	13 (68.4)	<0.001
	Gentamicin	126	13 (10.3)	5 (4.9)	8 (34.8)	<0.001
	Tobramycin	119	29 (24.4)	17 (17.0)	12 (63.2)	<0.001
Penicillins		126	101 (80.2)	78 (75.7)	23 (100.0)	0.008
Cephalosporins	Ceftaroline *	119	0 (0.0)	0 (0)	0 (0.0)	
Fluoroquinolones		126	29 (23.0)	12 (11.7)	17 (73.9)	<0.001
	Ciprofloxacin	126	28 (22.2)	11 (10.7)	17 (73.9)	<0.001
	Levofloxacin	119	24 (20.2)	9 (9.0)	15 (78.9)	<0.001
	Moxifloxacin	22	22 (100.0)	7 (100)	15 (100)	
Lincosamides	Clindamycin	126	39 (31.0)	28 (27.2)	11 (47.8)	
Macrolides	Erythromycin	125	47 (37.6)	31 (30.1)	16 (72.7)	<0.001
Streptogramins	Quinupristin–dalfopristin	119	1 (0.8)	0 (0.0)	1 (5.3)	0.021
Oxazolidinones	Linezolid *	126	0 (0.0)	0 (0.0)	0 (0.0)	
Tetracyclines		126	10 (7.9)	6 (5.8)	4 (17.4)	
	Minocycline	118	1 (0.8)	0 (0.0)	1 (5.3)	0.022
	Tetracycline	126	10 (7.9)	6 (5.8)	4 (17.4)	
Phenicols	Chloramphenicol	119	2 (1.7)	1 (1.0)	1 (5.3)	
Lipopeptides	Daptomycin *	119	0 (0.0)	0 (0.0)	0 (0.0)	
Phosphonic acids	Fosfomycin	118	1 (0.8)	0 (0.0)	1 (5.3)	0.022
	Mupirocin	126	22 (17.5)	13 (12.6)	9 (39.1)	0.002
	Pristinamycin *	119	0 (0.0)	0 (0.0)	0 (0.0)	
Ansamycins	Rifampicin	126	1 (0.8)	0 (0.0)	1 (4.3)	0.034
Folate pathway inhibitors	Trimethoprim–sulfamethoxazole *	126	0 (0.0)	0 (0.0)	0 (0.0)	
Glycopeptides and lipoglycopeptides	Teicoplanin *	119	0 (0.0)	0 (0.0)	0 (0.0)	
	Vancomycin *	119	0 (0.0)	0 (0.0)	0 (0.0)	
Olive leaf extract	OLE 25% *	126	0 (0.0)	0 (0.0)	0 (0.0)	
	OLE 12.5% *	126	0 (0.0)	0 (0.0)	0 (0.0)	
	OLE 6.25% *	126	0 (0.0)	0 (0.0)	0 (0.0)	
	OLE 3.125%	126	119 (94.5)	99 (96.1)	20 (87.0)	

Note. Data are expressed as absolute (N) and relative (%) frequencies for categorical variables. *p* values from the Χ^2^ test for significant differences in the proportion of antibiotic resistance strains between MSSA and MRSA are shown for each antibiotic. * Antibiotics to which all strains tested were susceptible. MSSA: methicillin-susceptible *Staphylococcus aureus*; MRSA: methicillin-resistant *Staphylococcus aureus*.

## Data Availability

The original contributions presented in this study are included in the article/Supplementary Material (Clinical strain characterisation is available in the Supplementary Material (Table S1), and the phenolic profile of OLE is available in the Supplementary Material (Table S2)). Further inquiries can be directed to the corresponding author.

## Supplementary Materials

The following supporting information can be downloaded at: https://www.mdpi.com/article/10.3390/ph18091358/s1, Table S1: Clinical strain characterisation. Table S2: Phenolic profile of OLE.

## Abbreviations

The following abbreviations are used in this manuscript:

SSTIs	Skin and soft tissue infections
MSSA	Methicillin-susceptible *Staphylococcus aureus*
MRSA	Methicillin-resistant *Staphylococcus aureus*
OLE	Olive leaf extract
MBC	Minimum bactericidal concentration
CFU	Colony-forming units
AMR	Antimicrobial resistance
UVB	Ultraviolet B
IQR	Interquartile range
DF	Diabetic foot
IS	Immunosuppression
PU	Pressure ulcer
SIWDD	Skin infection with dermatological disease
SIWODF	Skin infection without dermatological factors
SP	Surgical procedure
VD	Vascular disease
ATCC	American Type Culture Collection
EUCAST	European Committee on Antimicrobial Susceptibility Testing
MIC	Minimum inhibitory concentration
CBA	Columbia blood agar
MALDI-TOF	Matrix-Assisted Laser Desorption/Ionisation Time of Flight
MHA	Müller-Hinton agar
CAMHB	Cation Adjusted Müller-Hinton Broth
SD	Standard deviation
UPLC-MS/MS	Ultra-Performance liquid chromatography/tandem mass spectrometry system
